# Miseren des Krankenhauses, institutionelle Pathologien und klinische Organisationsethik

**DOI:** 10.1007/s00481-021-00628-z

**Published:** 2021-04-12

**Authors:** Matthias Kettner

**Affiliations:** Fakultät für Wirtschaft und Gesellschaft, Universität Witten/Herdecke, Alfred-Herrhausen-Straße 50, 58448 Witten, Deutschland

**Keywords:** Organisationsethik, Moralverantwortung, Sozialpathologie, DRG-System, Kommerzialisierung, Organizational ethics, Moral responsibility, Institutional pathology, DRG (Diagnosis-related groups), Commercialization

## Abstract

Personal und Patienten in Einrichtungen organisierter Krankenbehandlung erfahren und bekunden vielfältige miserable Zustände dieser Organisationen. Einige „Miseren“ lassen sich im Rahmen einer Theorie institutioneller Pathologien als störende Auswirkungen der Aktivitäten und Strukturen von Organisationen des Politiksystems (Gesundheitspolitik) und des Wirtschaftssystems (Gesundheitswirtschaft) erklären. Deshalb können Klinische Ethik-Komitees (KEKs) solchen Miseren nicht wirksam begegnen oder sie sogar nicht einmal thematisieren. Organisationsethik kann sie thematisieren, aber ihnen nicht wirksam begegnen. Vorgeschlagen wird die Verstärkung von Organisationethik durch eine Theorie institutioneller Pathologie. Institutionspathologisch aufgeklärte, „klinische“ Organisationsethik kann helfen, in Ethiktheorie und Praxis, etwa für Mitglieder von KEKs und Organisationethikberater, die Möglichkeiten der Beobachtung, Bewertung und ggf. Besserung von gestörten Verantwortungsverhältnissen erheblich zu erweitern.

## Abstract (en)

**Definition of the problem:**

Staff and patients in institutions of organized health care experience and express a variety of adverse conditions of these organizations. Within a theoretical framework of institutional pathology we can explain some of these “miserable conditions” as effects of the activities of organizations belonging to the political system (health policy) and to the economic system (health economy). Clinical ethics committees (CECs) cannot effectively handle such adversities or even address them properly. Standard organizational ethics can address them but cannot handle them effectively.

**Arguments:**

I propose to strengthen organizational ethics by a theory of institutional pathology. Basic nosological distinctions such as disease, illness and sickness, whose primary reference is to biological organisms and persons, can be analogically extended to socially constituted entities (e.g. hospitals) in terms of functional deficiency, miserable conditions, and need for reform of such entities. A very promising focus for analysis are organizational disorders of responsibility allocation. This group of institutional pathological disorders engenders, amongst other kinds of miserable conditions, specifically morally relevant miserable conditions.

**Conclusion:**

The institutional pathology paradigm, oriented to concrete institutions and organizations, can help “clinical” organizational ethics to considerably expand, in theoretical ethics as well as in ethically guided practice e.g. for members of CECs and organizational ethics advisors, the capacities for observing, evaluating and, if necessary, amending disturbed relations of responsibility.

## Warum überhaupt Organisationethik?

In jedem Einzelfall organisierter Krankenbehandlung hängt die ethische Qualität von weit mehr ab als nur dem guten Willen und Können der unmittelbar beteiligten Personen. James Evan Sabin, der Direktor des Harvard Pilgrim Health Care Ethics Programs, bringt es auf den Punkt: „The ethical quality of medical care depends as much on the ethics of organizations as the ethics of individuals. For better and worse, the culture and policies of hospitals, group practices, insurers, and other health system organizations shape individual clinician-patient relationships. We can’t have ethical healthcare without ethical organizations!“ (Sabin 2016, S. 111). Die Verbreitung dieser Einsicht steht im Hintergrund eines auch hierzulande wachsenden Interesses an Theorie und Praxis von Organisationsethik (OE).^1^

Institutionsformen von Ethikberatung und Klinische Ethikkomitees,^2^ an die sich die Organisationskultur des Gesundheitswesens mittlerweile gewöhnt hat, sind nach Wissensbasis, Kompetenzen und Zuständigkeiten zu begrenzt für die Komplexität organisationsethischer Probleme. Diese entstehen an den Spannungslinien zwischen mehr oder weniger gut begründeten Belangen der professionellen ärztlichen Behandlung, mehr oder weniger gut begründeten wirtschaftlichen Belangen und mehr oder weniger gut begründeten Belangen von Verwaltung und Management innerhalb einer Organisation und in den Beziehungen zu den anderen Organisationen, mit denen sie funktional verkoppelt ist. Genauer gesagt: Wenn miserable Zustände in einer Organisation zum Problem werden, dann zeigen sich diejenigen Problemaspekte, zu deren Lösung speziell Organisationsethik etwas beitragen können sollte, sobald man auf die „moralischen Kosten“ d. h. auf moralisches Unrecht achtet, das durch Reibungen zwischen diesen verschiedenartigen Belangen entsteht. Sobald vielfältige (ärztliche, pflegerische, volks- und betriebswirtschaftliche, administrative, politische) Ethosrationalitäten^3^ im Spiel sind, muss die kritische ethische Urteilsbildung^4^ mit Hierarchisierungskonflikten zwischen diesen rechnen und Formen der moralisch integren Kompromissbildung und Kompensation entwickeln. Wenn Organisationsethik proaktiv sein will, muss sie zudem moralisch zielführende organisationale Lernprozesse anregen, hierfür zweckdienliche Reorganisationsvorschläge entwickeln und Widerstände gegen deren Verwirklichung analysieren können und kritisieren dürfen. Diese anspruchsvollen Aufgaben erfordern einen großen Werkzeugkasten, dem die folgenden Überlegungen das Instrumentarium einer Theorie gestörter Institutionen hinzufügen wollen. Dieser Versuch ist neuartig, kann hier nur im Grundriss dargestellt werden und mutet den Leser*innen eine Reihe ungewohnter Begriffe und Denkfiguren zu, die aus gewohnten Sprachspielen von Organisationsberatung und Krankenhausmanagement ausscheren – allerdings mit dem durchaus praktischen Fernziel, bereichert zu diesen zurückzufinden.

## Warum überhaupt Organisationethik?

Im Hinblick auf Krankenhäuser und verwandte Einrichtungen, deren Existenz sich letztlich aus dem Allgemeininteresse rechtfertigt, dass gesundheitlich beeinträchtigte Menschen fachkundige Hilfe in Anspruch nehmen können, scheinen wir ein einfaches normatives Priorisierungsprinzip zu haben: Um die Aktivitäten von Organisation vom Typ eines Krankenhauses moralisch zu beurteilen, müssen Maßstäbe *klinischer* Ethik^5^ die ranghöchsten sein, denn es geht ja um Einrichtungen der Krankenbehandlung. Doch ganz so einfach ist es nicht. Das normativ Ranghöchste ist nicht auch schon das normative Ganze. In den hoch arbeitsteiligen Krankenhäusern und anderen verwaltungstechnisch komplexen Einrichtungen des Gesundheitswesens entstehen oft hartnäckige Probleme aus Miseren, deren moralische Aspekte die anwendungsorientierte Ethik vor Herausforderungen stellen, die weder die übliche, auch nach 40 Jahren noch auf einige wenige prima facie Prinzipien („Georgetown-Mantra“) fixierte Bioethik traktieren kann (Beauchamp und Childress 2019), noch allein die auf die inneren Werte der Arzt-Patient-Interaktion fixierte ärztliche Standesethik.

Auf die lange Liste solcher moralisch problembesetzter Miseren gehören: Unter‑, Über- und Fehlversorgung von Patientinnen und Patienten, Ausbeutung, Demotivierung und Diskriminierung von Personal, Ineffektivität, Ineffizienz, Korruption, Irreführung von Öffentlichkeit oder Behörden, Unaufrichtigkeit begünstigende Vergütungsverfahren, Wagenburgmentalität und Feindbilder, Nepotismus, Seilschaftenbildung, Egomanie auf Kosten von Organisationszielen, Konfundierung persönlicher und professioneller zwischenmenschlicher Beziehungen, Verdinglichung von Personen (etwa durch Stereotypisierungen), das Verleugnen von Missständen, Unterdrückung von Kritik, Überverrechtlichung, Überbürokratisierung, Überteuerung, Bereicherung (vgl. Kettner 2011).

Reibungen zwischen verschiedenartigen Belangen auf der Ebene der* konkreten Organisation* selbst (z. B. Reibungen zwischen administrativen Belangen des Datenschutzes und betriebswirtschaftlichen Belangen der Sparsamkeit im privatisierten Krankenhaus XY) erscheinen bei Betrachtung der Handlungsebene als Schwierigkeiten in den verschiedenartigen *Beziehungs- und Interaktionsformen von Personen, *nämlich derjenigen, die das Personal und die Klientel der Organisation bilden. Ihre Beziehungs- und Interaktionsformen sind aufgrund der unterschiedlichsten Rollendefinitionen innerhalb der konkreten Organisation an unterschiedlichen Funktionsstellen ganz unterschiedlich normiert. Die konkrete Organisation als Ganze aber ist die Einheit, die die Beziehungs- und Interaktionsformen aller Personen überformt und koordiniert. Man denke an die Beziehungen zwischen und innerhalb professionalisierter Berufsgruppen, z. B. zwischen ärztlichem und Pflegepersonal, aber auch zwischen Hierarchiestufen innerhalb des ärztlichen und innerhalb des Pflegepersonals. Man denke an Beziehungsformen des Lernens und Lehrens (z. B. die Aus- und Weiterbildung von Personal oder Studierenden) oder an Beziehungsformen in der medizinischen Forschung (z. B. Teams drittmittelgeförderter Forschung in Universitätskliniken). In allen arbeitsteiligen formalen Organisationen (z. B. in Wirtschaftsunternehmen und Verwaltungen) sind Beziehungsformen vertragsmäßiger Art hoch bedeutsam (Donaldson 2009). Denn solche Beziehungen erzeugen und garantieren Rechte und Pflichten (z. B. Rechte von Ärzten und Patienten im Beschäftigungsverhältnis und im Behandlungsvertrag; die Rechte und Pflichten von Produzenten und Konsumenten in marktwirtschaftlichen Unternehmen; die Rechte und Pflichten weisungsbefugter Vorgesetzter und ihrer Untergebenen in bürokratischen Einrichtungen).

## Miseren der DRG-Transformation

Vertragsbeziehungen sind besonders geeignet, um ökonomische und politische Vorgaben, Kräfte, Standards (z. B. Vergütungsvorgaben, Sparzwänge, Rentabilitätsstandards) gewissermaßen in den organisationalen Innenraum der Krankenversorgung zu übersetzen. Solche Vorgaben, Kräfte, Standards kommen z. B. in den Makro-Allokationsentscheidungen der Gesundheitspolitik und den Kostenkontroll-Politiken der Versicherungsträger zum Ausdruck.

Die Umstellung der klinischen Leistungsvergütung auf ein DRG-System in Deutschland ist ein Musterbeispiel solcher Übersetzungen. Das DRG-System war ursprünglich nur ein Kostenkontroll-, aber kein Vergütungssystem, wird aber in seiner in Deutschland seit 2003 eingeführten Form (G-DRG) von der Gesundheitspolitik mit dieser Funktion ausgestattet. Es führt in Wechselwirkung mit einer systematischen Untererfüllung der Verantwortung der öffentlichen Hand, Investitionskosten für Krankenhäuser zu übernehmen, zur Finanzmisere vieler Krankenhäuser in nichtprivater Trägerschaft. So entsteht ein starker Einsparungsdruck, den die betriebswirtschaftliche Führung dieser Häuser dann verarbeiten muss und – im Verständnis guter betriebswirtschaftlicher Verantwortung – oft auch nur so verarbeiten kann, dass die medizinethisch gebotene Vorrangstellung des ärztlich verstandenen Patientenwohls beeinträchtigt wird (Arbeitsgruppe „Ökonomisierung“ 2020).

Wechselwirkungen des funktionspervertierten (nämlich administrative Belange mit betriebswirtschaftlichen Belangen überfrachtenden) DRG-Systems, für das konkrete Organisationen der Gesundheitspolitik verantwortlich sind, mit – ebenfalls politisch seit langem geförderten – starken Interessen von marktwirtschaftlichen Organisationen an der Umstellung des Gesundheitssystems auf Gesundheitswirtschaft haben einen erheblichen Anteil an vielen der problembelasteten Miseren, die von den Leidtragenden zunehmend beklagt (Stern 2019) und, freilich oft allzu pauschal, als „Ökonomisierung des Gesundheitswesens“ kritisiert werden.^6^

## Vier Konzeptionen von Organisationsethik

Wie müsste OE theoretisch aufgestellt sein, die der Komplexität von inter- und intrasystemischen problemerzeugenden Wechselwirkungen gerecht werden könnte, statt Moralprobleme, wie sie innerhalb einer konkreten Organisation auftauchen, bestenfalls nur unter den vorgegebenen Funktionsbedingungen dieser Organisation zu identifizieren und zu beurteilen? Anders gefragt: Wie kann OE kritisch und reformorientiert sein?

Mindestens vier deutlich verschiedene Konzeptionen von OE haben sich bislang herausgebildet (vgl. Krobath 2010, S. 553): OE als (1) Management-Ethik, als (2) Leitbildethik, als (3) Ethik konkreter individuierter Organisationen, und OE als (4) Praxis des „Organisierens der Ethik in Organisationen“ (Krobath 2010, S. 556). Im Handbuch von Krobath und Heller dominiert die vierte Lesart, „die Organisation ethischer Reflexion als dauerhaft zu erneuernder Prozess in Organisationen“ (Krobath und Heller 2010, S. 19).^7^

Diese vierte, interventionspraktische Lesart hat gewiss viel für sich. Warum? Kurz gesagt: Soweit OE normativ wird, also über die Verbesserung von Tatsachenwissen hinaus auch begründete (Um)Wertungen und (Re)Normierungen anstrebt, ist sie eine Gestalt angewandter normativer Ethik. In ihrer Anwendung ist normative Ethik aber keine freistehende und kontemplative, sondern eine engagierte und transformative Praxis. Die interventionistische Verwendung moralischen Denkens ist eine Form der Ausübung diskursiver Macht^8^ und somit ein praktisches Verhältnis, kein bloß theoretisches. Angewandte Ethik ist intervenierend, sobald man versucht, normative Überzeugungen moralischer Art (z. B. Moralprinzipien aus philosophisch ausgewiesenen Positionen wie Utilitarismus, Kontraktualismus, Kantianismus, „Principles of Biomedical Ethics“ usw.) in bestimmten Praxisbereichen geltend zu machen, um gewisse Problemlagen, die dort typisch anfallen und eine moralisch irritierende Seite haben, besser zu bewältigen, und zwar in einem moralisch qualifizierten Sinne von „besser“. Die Tatsache, dass die moralische Verbesserung von als problematisch wahrgenommenen Praktiken das Kerngeschäft angewandter Ethik ist, macht diejenigen, die es betreiben, gewissermaßen zu Problembetroffenen zweiter Ordnung. Wir machen Moralprobleme, die die Menschen in der betreffenden Praxis haben, virtuell zu unserem Moralproblem, etwa wenn wir mit organisationsethischem Mandat bestimmte Veränderungen anraten. Daher sollte wenigstens die *Theorie* dieser Interventionspraxis den Eigensinn dessen, was sie als „moralische Probleme“ konstruiert, gut zu klären versuchen. Denn durch ein bloß mangelhaft aufgeklärtes Problembewusstsein erhöht sich die Gefahr, Probleme, die bei Licht besehen keine moralischen sind, für solche zu halten und als rein moralische zu behandeln – Gefahren des „Moralismus“ –, und umgekehrt Probleme, die (zumindest vonseiten der direkt Betroffenen) als moralische aufgefasst werden, nicht als solche zu behandeln, sondern z. B. als rein technische, psychologische, administrative, manageriale, wirtschaftliche etc. – Gefahren der „Moralverdrängung“. Und gewiss muss gut organisierte OE auch so organisiert sein, dass der Spielraum ihrer Reflexionsmächtigkeit auch dann noch operativ nachhaltig Beachtung finden kann, also etwas ausrichten kann, wenn sie nicht einfach nur die in Betrieb befindlichen Funktionen und schon eingerichteten Abläufe, nicht einfach nur den lokal vorfindlichen Institutionalisierungsstand diverser Ethosrationalitäten bejaht, sondern auf Distanz geht, die Abläufe unterbricht (Heintel 2010, bes. S. 460) oder irritiert und dadurch Widerspruch (Krainer 2010) und Konflikte erzeugt.

Die dritte Konzeption, OE verstanden als auf konkrete individuierte Organisationen und Organisationstypen angewandte Ethik,^9^ hat vor der vierten einen sachlichen Vorrang oder bildet zumindest eine notwendige Ergänzung. Denn bevor man versucht, Ethik („Ethikreflexion“) in eine Organisation hineinzubringen, muss man explorieren, wieviel an Responsivität für moralische Irritationen und was an institutionalisierten Ethosrationalitäten in der betreffenden Organisation bereits vorhanden ist (z. B. in einer privatisierten Universitätsklinik: ein ärztliches Professionsethos, ein pflegerisches Professionsethos, ein unternehmerisches Ethos, ein akademisches Ethos), welche Konfliktlinien diese unterschiedlichen Ethosrationalitäten aufspannen und wie solche Konfliktlinien sich verändern, wenn auffällige, erklärungsbedürftige Verantwortungsstörungen die Organisation plagen (z. B. infolge von überwertiger Ökonomisierung bzw. Kommerzialisierung^10^).

## Form und Inhalt der Moral als Gegenstand von OE

Eine eklatante Schwäche in der OE-Literatur zu allen vier Typen ist die oft im inhaltsleeren Verweis auf „Werte“, „Ideale“, „den moralischen Gesichtspunkt“ abstrakt und formal bleibende Rede von Moral. Wer den Bezug zur Moral inhaltlich unbestimmt lässt, gewinnt scheinbar eine vorteilhafte Offenheit gegenüber vielfältigsten Moralvorstellungen. Jedoch: Wird der Bezug auf Moral inhaltlich unbestimmt gelassen, kann nicht mehr angeben werden, wodurch ein erkennbar relevantes Problem zu einem erkennbar moralisch relevanten Problem wird. Das führt, so seltsam es klingt, zu moralblinder OE.

Einen m. E. besseren theoretischen und daher auch für OE praktisch besseren Umgang mit moralischer Diversität, der Einheit und Vielfalt von einbekannten Moralvorstellungen, bietet eine interpretationsoffene Strukturanalyse von Verantwortung. Für alles moralisch qualifizierbare Verhalten ist eine entsprechende Wahrnehmung von Verantwortung unabdingbar. Als notwendig und hinreichend dafür, es bei einem Problem mit einem moralischen Problem zu tun zu haben, zählt dann die normative Überzeugung, jemandem (oder etwas, das für uns als Objekt von Rücksichtnahme moralintern zählt bzw. einen gewissen „moralischen Status“ hat) geschehe durch bestimmte Aktivitäten seitens bestimmter Akteure (das können natürliche Personen oder auch korporative Akteure sein) *etwas Unrechtes, das eigentlich nicht sein soll. *Die Leitunterscheidung in der konkreten allgemein verbreiteten Moral („Common Morality“, Gert 1998, 2007) ist die adverbiale Unterscheidung von *unrechtem/nicht-unrechtem* Verhalten von Akteuren, die für uns als gründe-responsive Akteure zählen, d. h. als verantwortungsfähige und vernunftfähige Akteure (vgl. Kettner 2014). Freilich ist es oft so, dass wir etwas Unrechtes, das eigentlich nicht sein soll, nicht sofort klar und unmittelbar erkennen, schon gar nicht in Situationen organisational vermittelten Handelns, etwa im multiprofessionellen Behandlungsteam innerhalb einer funktional hoch ausdifferenzierten Organisation. Subjektive Eindrücke des Fragwürdigen, Gefühle des Unbehagens, Empfindungen des Verletztseins müssen erst artikuliert und geklärt, diffuse Verantwortungszuschreibungen müssen sondiert, Problemdefinitionen müssen sortiert werden, usw. Der hier anfallende Reflexionsbedarf liefert den vielleicht besten Rechtfertigungsgrund für die Existenz z. B. von klinischen Ethik-Komitees (vgl. Kettner 2008).

Die konkrete allgemein verbreitete Moral enthält keineswegs nur jene starken normativen Bewertungsgründe, deren Sinngehalt wir auch als allgemeinverbindliche Moralregeln ausdrücken können (z. B. „neminem laede!“, „betrüge niemanden!“, „erfülle deine Verpflichtungen!“, „halte deine Versprechen!“ u. a. m.). Vielmehr bildet unsere Moral ein reichhaltiges normatives Netzwerk verschiedenartiger Gründe. Das kann man sich so klarmachen: Die Anerkennungswürdigkeit jeder bestimmten allgemeinverbindlichen Regel mit sozusagen moralischem Ausrufezeichen hat für uns stets ihre überzeugenden „Hintergründe“, die den Geltungsanspruch der Regel fundieren und ihre Autorität aus unserer Sicht rechtfertigen. Die philosophische Ethik hat eine Reihe von starken Hintergrundpositionen entwickelt (u. a. Utilitarismus, Kontraktualismus, „Kantianismus“). Doch ebenso wichtig ist in der konkreten allgemein verbreiteten Moral eine weitere Art guter Gründe, die wir „Entschuldungsgründe“ nennen könnten: Z. B. würden wir das Abweichen von einer bestimmten Moralregel in einer konkreten Situation (z. B. das Abweichen von der allgemein fest begründeten Regel der ärztlichen Wahrhaftigkeit in besonderen Situationen sorgsam schonender Aufklärung) nur dann als moralisch falsch verurteilen, wenn in der konkreten Situation, wohl verstanden, keine guten Gründe liegen, die uns, falls alle Betroffenen sie nur unvoreingenommen würdigen könnten und wollten, den Regelbruch im konkreten Fall als *frei* von Unrechtem, das nicht sein soll, begreifen lassen. Z. B. wäre in einer akuten Situation der abhilfelosen Knappheit von notwendigen Behandlungsmitteln die planvolle Nichtversorgung einiger Schwerverletzter, die die geringsten Überlebenschancen haben, und Versorgung anderer, die bessere Überlebenschancen haben, professionsethisch betrachtet ein massives moralisches Unrecht, wenn wir nicht gelernt hätten, *auch* die in dieser Situation liegenden guten Entschuldungsgründe angemessen zu würdigen. Das Stichwort Triage steht für einen moralisch belangvollen Lernprozess.

Aus unzähligen Beispielen unserer Lebenserfahrung, die vom Dramatischen bis ins Banalste herunterreichen, erhellt: Moralische Normativität („unsere Moral“), kulturell verkörpert im informellen Netzwerk von (1) allgemein anerkannten Moralregeln, (2) autorisierenden Hintergründen und (3) überzeugenden Entschuldungsgründen, ist kein starres, sondern ein lernfähiges, an sich wandelnde Umstände unserer Lebenswirklichkeit sich intelligent anpassendes Orientierungssystem. OE arbeitet immer, freilich je nach Konzeption auf verschiedene Weise, mit dem, was aus diesem Orientierungssystem werden kann und werden sollte unter den modifizierenden Kräften der „Lebenswirklichkeit“ einer konkreten Organisation.

## Verantwortungsverhältnisse als angemessene Moralperspektive für OE

Spezifisch moralisch relevant werden miserable Organisationszustände genau in dem Maße, wie Veränderungen in den betreffenden Zuständen zugleich Veränderungen in der Bilanz von unentschuldeten Verletzungen anerkennungswürdiger moralischer Regeln machen. Anders gesagt: In dem Maße, wie miserable Organisationszustände tatsächlich zu Unrecht, das nicht sein soll und nicht sein müsste, führen oder dessen Eintreten erheblich wahrscheinlicher machen. Wirkungsvoll ist OE als Anwalt moralischer Probleme und Problemlösungen nur dann, wenn sie durch ihre Interventionen *diese* Bilanz nachweislich verbessert, und nicht etwa (nur) die Betriebsbilanz oder die durchschnittliche Patientenzufriedenheit. So sieht es auch Sabin (2016, S. 115, meine Hervorhebung, M. K.): „The ultimate aim and justification for organizational ethics activities is to make *an ethically meaningful difference* in how an organization functions. Unless that happens, the activity will be merely academic, and likely to lead to frustration. The effort to make a difference requires managerial skill on the part of the clinical ethics committee, strong relationships with organizational leaders, and practical understanding of what kinds of outputs will be valued and used by the organization.“

Die moralischen Überzeugungen der einen decken sich manchmal mehr und manchmal weniger mit denen der anderen. Die moralische Diversität kann gerade in Situationen der Krankenbehandlung dramatisch sein. Was im Licht einer professionell ärztlichen Ethosrationalität eine lebensrettende Blutinfusion wäre, deren Unterlassung moralisch massiv unrecht wäre, mag sich im Licht einer anderen Ethosrationalität (z. B. einer religiös begründeten) als ein höchst unheilvolles moralisches Unrecht darstellen. Solche Divergenzen entspringen der Einbettung unserer konkreten allgemein verbreiteten Moral in unterschiedliche Ethosrationalitäten und sind mit ihrem Anspruch auf Allgemeinverbindlichkeit keineswegs unvereinbar. Divergenzen machen allerdings deutlich, dass im Rahmen unterschiedlicher Ethosrationalitäten erheblicher Spielraum für die Modulation der Regeln und Ideale der Allgemeinmoral besteht. Vernünftige Ethik, ergo auch vernünftige OE, muss solchem Spielraum gerecht werden. Es hilft wenig, am Begründungsende der Regeln der Allgemeinmoral auf eine bestimmte Rechtfertigungslehre sich zu fixieren, wenn auf der intellektuellen Höhe philosophischer Rechtfertigungslehren selbst zuletzt doch kein Monopol herausvernünftelt werden kann.^11^ Aus dieser Einschätzung lässt sich eine für die Theoriekonstruktion von OE hilfreiche Einsicht gewinnen:

Hinreichend offen wird „die Moralperspektive“ in der Ethik, also auch in der OE, erst dann begriffen, wenn damit nicht mehr und nicht weniger gemeint ist als eine Fähigkeit, die in der Kommunikations- und Interaktionsgemeinschaft von Menschen typischer- und normalerweise entwickelt wird: Die Fähigkeit, (1) repräsentativ ernstzunehmen, wie (2) intentional kontrollierbare Aktivitäten (3) in bestimmten Bereichen, für die bestimmte Akteure als zuständig gelten, (4) zum Guten oder Schlechten (5) all der Personen (und u. U. auch Tiere und anderer „Moralobjekte“) ausschlagen, die diesbezüglich für uns zählen sollen – all dies immer interpretiert im Licht je maßgeblicher Ethosrationalitäten. Die so strukturierte komplexe Fähigkeit können wir einfachheitshalber als *Mitbetroffenheit* bezeichnen und meinen damit die Fähigkeit, Verantwortung moralisch-normativ zu interpretieren und sie praktisch wahrzunehmen. Fast alle Personen können das, viele korporative Akteure, z. B. Organisationen mit lernfähigen Leitungsstrukturen, können es ebenfalls (vgl. Neuhäuser 2011).

## Zum Ansatz einer Theorie institutioneller Pathologien

Im Rest dieses Aufsatzes möchte ich folgenden Vorschlag untermauern: Als eine auf konkrete individuelle Organisationen und Organisationstypen angewandte Ethik sollte OE die Diagnose, Erklärung und Besserung von Störungen in Organisationen in ihre Agenda aufnehmen, und zwar vordringlich von solchen Störungen, die die Wahrnehmung von Verantwortung moralischer Art systematisch und negativ folgenreich beinträchtigen, wobei die Gründe für die Entstehung oder Aufrechterhaltung der Störung im „Leben“ der Organisationen selbst liegen und nicht, oder jedenfalls nicht allein, in der persönlichen Lebensführung der Personen, die ihr Personal und ihre Klientel bilden. Wenn wir den Störungsbegriff ernstnehmen wollen, erscheint eine Erweiterung von OE um eine Theorie sozialer, genauer: institutioneller Pathologien angezeigt. Der gute Sinn einer solchen Theorie läge darin, vom Raffinement des in Medizin und Psychologie entwickelten Krankheits- und Störungsdenkens sachhaltig und einsichtsvoll etwas auf aktive organisierte Sozialgebilde zu übertragen und dies für OE nutzbar zu machen, nicht zuletzt um Erfolgserwartungen an intervenierende OE realistisch(er) abschätzen zu können. Gewiss, man ist gut beraten, die Chancen für wünschenswerte moralisch relevante Prozesse des Lernens und Umlernens in der „Organisationskultur“ zu suchen. Doch was OE mit bestimmten Interventionen, Denkanstößen, Change-Prozessen etc. ausrichten möchte und eventuell anrichtet, wird nicht zuletzt davon abhängen, wie und wie sehr die Organisation und ihre „Kultur“ gestört sind. Entsprechendes Wissen würde OE umsichtiger machen und insofern: verbessern.

Wenn diese anvisierte Erweiterung der Agenda konkreter OE einen eigenen Namen haben muss, wäre „klinische OE“ vielleicht eine passende Bezeichnung, weil es um Diagnose, Erklärung und Besserung von Störungen geht, wenngleich von Organisationen.^12^ Dieser Perspektivenwechsel ist verblüffend, aber kein Bluff. Eine wachsende Forschungsliteratur beschäftigt sich mit dysfunktionalen Organisationen, vor allem Wirtschaftsunternehmen.^13^ Auch in Diskursen der Sozialphilosophie und Sozialpsychologie gewinnt das Thema „sozialer Pathologien“ zunehmend Aufmerksamkeit.^14^ Die Frage, wie wir massiv gestörte Sozialgebilde theoretisch begreifen können, ist nicht nur wissenschaftlich relevant, sondern auch praktisch und politisch von hohem Interesse, da die theoretische Diagnose von Störungen unweigerlich auf die praktische Frage nach ihrer Behebbarkeit führt.

Als „institutionelle“ Pathologien möchten wir Pathologien bezeichnen, deren Genese (Ätiologie) und u. U. auch Erscheinungsweise (Symptomatik) nicht in den biotischen und mentalen Prozessen natürlicher Personen und anderer Lebewesen zu suchen sind, sondern in den funktional eingerichteten soziokulturellen Prozessen und deren Produkten, den sinnvollen Praktiken, aus denen aktive organisierte Sozialgebilde bestehen. Zu den drei nosologischen Hauptkategorien der objektivierbaren Krankheit (*disease*), der leidvoll erlebten Beeinträchtigung (*illness*) und der Behandlungsbedürftigkeit (*sickness*) schlagen wir als sozialtheoretische Entsprechungen die Kategorien der Funktionsdefizienz, der Misere und der Reformbedürftigkeit vor (vgl. Jacobs und Kettner 2016).

Zur Erläuterung: Der Allgemeinbegriff „Misere“ mag befremdlich klingen, taugt aber gut, denn egal ob man an wirtschaftliche, geschäftliche, finanzielle, politische, moralische etc. Miseren denkt, stets ist ein unglücklicher und auf je spezifische Weise elender Zustand gemeint. Der etwas sperrige anmutende Terminus „Funktionsdefizienz“ verweist in der deutschen genau wie in der englischsprachigen Fachterminologie (vgl. *functional deficiency*) auf fehlerhafte, ausfallende oder versagende, schlecht oder störanfällig laufende, in ihren „erforderlichen“ Leistungen mangelhafte, in ihren Wirkungen von ihren „normalen“ Zielwerten ungut abweichende oder mangelhaft aufeinander abgestimmte Prozesse innerhalb der Gesamtaktivität einer Einheit, die als ein Ganzes integriert und, eingelassen in eine Mit- und Umwelt, sich selbst erhaltend organisiert ist (wie bei natürlichen Organismen) oder sein sollte (wie bei technisch oder sozial gestalteten Artefakten, z. B. Organisationen).^15^

Damit produktive Anleihen bei psychologischen und medizinischen Krankheits- und Störungstheorien sachhaltig werden und nicht im Metaphorischen steckenbleiben, müssen kausal relevante Mechanismen ausfindig gemacht werden: Erstens solche, die bestimmte Funktionsdefizienzen in den betreffenden aktiven Sozialgebilden hervorrufen, aufrechterhalten oder veränderungsresistent machen. (Mit Blick auf das anfangs skizzierte Beispiel der DRG-Miseren wäre einer dieser Mechanismen die mittels einer gesundheitspolitischen Kausalkette bewirkte Kopplung der Funktion, gemäß ärztlichen Indikationen zu therapieren, mit unternehmerischen Funktionen). Zweitens solche kausal relevanten Mechanismen, die allererst machen, dass aus Funktionsdefizienzen Miseren werden, Miseren der betreffenden Sozialgebilde selbst (in Kliniken z. B. miserable Organisationszustände wie übermäßige Mitarbeiterunzufriedenheit und Personalfluktuation) oder auch Miseren in weiteren Sozialgebilden, die miteinander funktional verkoppelt sind.

## Zur Modellierung institutioneller Pathologien

Der Ansatz bei institutionellen Pathologien erlaubt die begriffliche Modellierung unterschiedlicher Störungsformen. Das kann hier nur schematisch angedeutet werden.

Das biomedizinische Grundmodell ist auf Einzelorganismen abgestimmt. In diesem finden Ätiologie, Symptomatik und Therapie ihren ontologischen Ort. In einem ersten, einfachen Störungsmodell der Institutionspathologie – nennen wir es M1 – erweitern wir nun den ätiologischen Ort und den therapeutischen Ort, aber noch nicht den symptomatischen Ort. Ein klassisches Beispiel für M1 war Freuds Diagnose, die bürgerliche Sexualmoral verursache massenhaft neurotische Störungen. Nota bene: Schon um 1900 sprachen einige Ärzte (z. B. Alfred Grotjahn) von „sozialer Pathologie“, um eine damals neue, nämlich ätiologisch erweiterte Betrachtungsweise von bereits wohlbekannten Krankheiten wie z. B. Tuberkulose unter „sozialen Gesichtspunkten“ zu bezeichnen. Der ätiologische Ort wurde um soziale „pathogene“ Gegebenheiten (z. B. unhygienische Arbeits- und Lebensbedingungen, oder, wie bei Freud, eine überrepressive, institutionalisierte Sexualmoral) erweitert. Die Logiken von institutionspathologischen und ärztlichen Diagnosen treten im Modell M1 noch nicht auseinander: Die Krankheit/Störung manifestiert sich an den Menschen. M1 bietet aber bereits einen guten Ausgangspunkt für institutionspathologische Forschungen. Das wird deutlich, sobald wir für jene „sozialen Verhältnisse“, die gemäß Modell M1 ein ätiologisches Ortsrecht erhalten, weil sie in M1 als pathogen (krankmachend) begriffen werden, modernere sozialtheoretische Konstrukte zulassen wie z. B. Mentalitäten, Dispositive, Diskurse, Narrative, Exklusionsstrategien, strukturelle Gewaltverhältnisse, soziale Ungleichheit u. a. m.

Ein gegenüber M1 angereichertes Modell, nennen wir es M1+, resultiert, wenn es gelingt, neue, überraschende Störungsdiagnosen und/oder neue leidvolle Beeinträchtigungen dingfest zu machen, die im *Disease*-Katalog der medizinisch anerkannten Krankheiten und leidvollen Beeinträchtigungen von Menschen (noch) nicht vorkommen, also lege artis noch nicht als Krankheiten und Krankheitssymptome „gelten“ (z. B. Selbstentfremdung, Enhancement-Sucht, Endgeräte-Abhängigkeit, Klimakatastrophen-Depression, oder mit Blick auf schwere Verwerfungen der öffentlichen Meinungsbildung in Zeiten von Covid-19 und Corona-Kontrollpolitik, womöglich *massive infosphere disorder* als eine neuartige Funktionsdefizienz im Mediensystem).

Wirklich interessante Erweiterungsmöglichkeiten ergeben sich, wenn wir aktive Sozialgebilde *als solche* zur Referenz von Krankheits- und Gestörtheitsurteilen machen: Modell M2. In pathologisch gestörten aktiven Sozialgebilden arbeiten wichtige innere und äußere Funktionen defizient, und zwar so, dass sich dies als Misere in allen oder in einigen der mit Recht erwartbaren Normalleistungen der betroffenen Sozialgebilde manifestiert. Aktive organisierte Sozialgebilde können sogar quasi todkrank sein – die Auflösung (z. B. die Auflösung einer heillos korrupten Klinik) wäre dann ein Äquivalent für den *exitus*.

Da man von Krankheit/Gestörtheit ohnehin nur bei massiven, jedenfalls nichttrivialen Störungen sprechen sollte, wäre Folgendes ein klarer Fall massiver Funktionsdefizienz: Sozialgebilde sind normalerweise offen für innovative oder reparative Um- und Neukonstruktionen, für Reformen und entsprechende Lernprozesse, wenn nur genug Menschen, die in die Aktivitäten der betreffenden Gebilde einbezogen sind (d. h. ihr Personal und Klientel, z. B. die Studierenden, Dozenten, Verwaltungsleute als Personal der Organisation Universität), diese Aktivitäten massiv „nicht mehr in Ordnung“ finden. Angenommen aber, bestimmte Funktionen wären so schwer gestört, dass mit den verfügbaren „Bordmitteln“ keine Abhilfe mehr geschaffen werden könnte, dann hätten wir einen klaren Fall von *massiver* Defizienz. Und wenn die Funktionsdefizienz so massiv wäre, dass eine Reform auch mit anderen Mitteln nicht mehr machbar wäre, würden wir von einem *hoffnungslosen Fall *sprechen. Bei Auflösung der betreffenden Sozialgebilde könnten wir durchaus vom soziokulturellen *Tod* sprechen. Organisationen können an den Komplikationen ihrer systemischen Krankheiten sterben.

Finden wir auch ein sozialpathologisches Äquivalent von *Illness, *also von Krankheitswertigkeit, leidvoller Beeinträchtigung? Sozialgebilde leiden gewiss nicht wie Menschen und Tiere, aber warum sollte es unmöglich sein, mit Hilfe von passenden, auf die Eigenart der jeweiligen Gebilde abgestimmten Werttheorien gewisse miserable Zustände, die aus bestimmten Funktionsdefizienzen entspringen, quasi als leidvolle Beeinträchtigungen *der betroffenen Sozialgebilde *zu begreifen? Wir können hier von Miseren der sozialen Gebilde selbst sprechen (statt nur vom krankheitsbedingten Leiden kranker Menschen, etwa von Teilen des Personals oder Klientels einer Organisation).

Anders als in M1 und M1+, treten in M2 die Logik von sozialpathologischen Diagnosen und die Logik von ärztlichen Diagnosen wirklich auseinander, denn gemäß M2 können Sozialgebilde *und* Lebewesen krank oder gesund sein, und dies auch unabhängig voneinander. Es kann gesunde, florierende Sozialgebilde mit gesundem Personal geben. Es kann kranke Sozialgebilde mit krankem Personal geben. Es kann kranke Sozialgebilde mit gesundem Personal geben und solche, die florieren, obwohl oder sogar weil sie mit gestörtem oder krankem Personal operieren. Zudem sind die Modelle M1 und M2 miteinander kombinierbar: Wenn zum Umfang der Misere eines gemäß Modell M2 kranken Sozialgebildes S auch solche Auswirkungen gehören, die gemäß Modell M1 als pathogene Auswirkungen zählen (= Auswirkungen, die für bestimmte Gruppen von Menschen die Wahrscheinlichkeit signifikant erhöhen, sich gesundheitliche Übel zuzuziehen), dann haben wir hier den Fall, dass pathologisch gestörte Sozialgebilde mittelbar oder unmittelbar Menschen krankmachen, d. h. krank im üblichen medizinischen Sinne.

Auch Modell M2 lässt sich noch anreichern: Im Modell M2+ soll begreiflich gemacht werden, dass Funktionsdefizienzen in S nicht an S selbst als Misere in Erscheinung treten, sondern so, dass S pathogen für andere Sozialgebilde S′ wird, die ihrerseits erst durch S funktionsdefizient („krank“) werden und in miserable Zustände geraten. (Ein klarer Fall von M2+ wäre eine Polizeibehörde, die von einer erfolgreichen mafiösen Organisation infiltriert wurde.)^16^.

## Pathologisch gestörte Verantwortungsverhältnisse: Ein Ausblick

Wie können wir Verantwortlichkeitsstörungen in Kliniken und verwandten Einrichtungen der organisierten Krankenbehandlung identifizieren, wie die Entstehung solcher Störungen und den Spielraum ihrer Veränderbarkeit institutionspathologisch erklären? Sicher sind die im vorigen Abschnitt skizzierten Modelle nur erst der Anfang eines Forschungsprogramms. Störungs- und Krankheitsmodelle verweisen umgekehrt auf Konzepte von Gesundheit. Vielleicht kann ein organisationsethisch gehaltvolles Konzept von Resilienz für die Theorie institutioneller Pathologien eine ähnliche Rolle spielen wie Gesundheitsbegriffe für die Medizin. Auch können wir einen organisationsethisch angepassten Begriff von moralischer Integrität kritisch auf Organisationen anwenden (Heubel und Kettner 2012). Fortschritt ist des Weiteren von der differenzierten Beschreibung unterschiedlicher Störungsbilder im Sozialpathologie-Paradigma zu erwarten. Wir haben mit den Verantwortungsverhältnissen bis jetzt ja nur ein einziges, für OE allerdings zentrales Störungsbild im Blick. Denn wie oben im Abschnitt über Verantwortung als angemessene Moralperspektive für OE ausgeführt, bilden Verantwortlichkeitsstörungen eine Gruppe von institutionspathologisch zu erklärenden Störungen, die für die OE konkreter Organisationen und Organisationstypen besonders wichtig sind und behandelt werden sollten. Genauer: Verantwortlichkeitsstörungen, die zur Entstehung, zur Aufrechterhaltung oder zur Hartnäckigkeit von moralisch bedeutsamen Miseren beitragen.

Verantwortung ist eine kombinierte Funktion von Wissen und Handlungsmächtigkeit; wer nichts weiß, ist so verantwortungsunfähig wie ein Ohnmächtiger. Unsere institutionspathologische Hypothese besagt zunächst, dass Verantwortlichkeitsstörungen aus Funktionsdefizienzen in denjenigen Prozessen entstehen, die die *Passung* von Macht (in diversen Formen) und von Wissen (in diversen Formen) regulieren, die sich in der Organisationsgeschichte einer konkreten Organisation an deren Entscheidungsstellen gebildet haben.^17^

Da von den Verantwortungsverhältnissen einer Organisation, wie ausgeführt, auch ihre Fähigkeit zur Mitbetroffenheit abhängt, besagt die organisationsethische Hypothese des Weiteren, dass Verantwortlichkeitsstörungen das Auftreten spezifisch von *moralisch relevanten *Miseren wahrscheinlicher machen und nicht etwa nur, wie betriebswirtschaftlich, juristisch und psychologisch zu vermuten wäre, die Vergrößerung von Insolvenzrisiken, Imageschäden, Gerichtsprozesse und hohe Personalunzufriedenheit.

Es wäre eine für die Theorie institutioneller Pathologien ergiebige Forschungsperspektive, durch Fallanalysen konkreter Organisationen zu prüfen, welche der vielfältigen Probleme, die sie plagen, als aus Funktionsdefizienzen hervorgehende Miseren sich erklären, bewerten und u. U. auch bessern lassen. Der Gewinn für „klinische“ OE läge in besseren Antworten auf die Frage, unter welchen Bedingungen und durch welche kausalen Mechanismen Verantwortlichkeitsstörungen entstehen und sich in moralisch miserable Organisationszustände übersetzen.

Gegen die hier vorgeschlagene „klinische“ Konzeption von OE mag man einwenden, dass sie zu kompliziert und spekulativ ist und zu weit außerhalb des Horizonts der Arbeit von Ethikkomitees und Organisationsberater*innen bleibt. Ich meine aber, dass gerade der steile Anspruch, empirische Organisationswissenschaft und Organisationsethik unter dem Bezugsproblem der Diagnose, Erklärung und Behandlung gestörter Institutionen zusammenzuführen, die Mühe wert ist. Er bietet faszinierende Aussichten. Aussichten auf eine Organisationsethik mit Biss.

## Footnotes

1 Für einen Überblick über 30 Jahre weitgehend angelsächsischer Literatur zur Organisationsethik im Gesundheitswesen siehe Suhonen et al. (2011). Interessant als Überbrückungsversuch zwischen der unternehmerischen Ethosrationalität und der Ethosrationalität von Ärzten ist immer noch Hall (2000). Repräsentativ für die vorwiegend deutschsprachige Literatur zur Organisationsethik im Gesundheits- und Wirtschaftsbereich ist das Handbuch von Krobath und Heller (2010).

2 Zur Verbreitung von Ethikberatung in Deutschland siehe https://ethikkomitee.de/index.html, zu Aufgaben und Verbreitung Klinischer Ethikkomitees siehe Woellert (2019), Neitzke (2009), Frewer et al. (2008).

3 Als „Ethosrationalitäten“ bezeichne ich normative Deutungsmuster, in denen maßgebende Überzeugungen des unrechtvermeidenden Sichverhaltens (Moral im engeren Sinne), maßgebende Überzeugungen des vernünftigen Sichverhaltens (Rationalitätsstandards) sowie hochgehaltene Ideale der Lebensführung eine kohärente Gestalt bilden (vgl. Kettner 2015).

4 Mit „Ethik“ und „ethisch“ meine ich eine über Tatsachenfragen hinausreichende Reflexion aller Norm- und Wertfragen, die für ein anerkanntes und gelebtes Ethos (gleich welchen Inhalts) relevant sind. Solche Reflexion kann mehr oder weniger implizit bleiben oder aber, wie in der philosophischen Ethik, systematisch sehr weit ausgebildet werden. Mit „Moral“ hingegen bezeichne ich ein spezifisches Feld innerhalb des weiten Untersuchungsbereichs der Ethik, nämlich alle anerkannten und gelebten normativen Überzeugungen von Regeln und Idealen (Gert 1998), die die Mitglieder kultureller Wir-Gruppen (idealiter wären das alle Mitmenschen) in einem einleuchtend begründbaren geteilten Verständnis von gemeinsamer Verantwortung (Mitverantwortung) für die Vermeidung unrechten Sichverhaltens voneinander erwarten (vgl. Kettner 2002, 2014) bzw. deren angemessene Beachtung sie einander schulden (Scanlon 2000).

5 „The relationship of patients with medically trained and licensed clinicians is at the very heart of CME [Clinical Medical Ethics]“ (Siegler 2019, S. 17).

6 Zur Präzisierung: „Ökonomisierungsprozesse lassen sich begreifen als systematische und durchgreifende Versuche, erwünschte Ergebnisse möglichst zielführend und möglichst sparsam zu erzielen. Solange Ökonomisierung sich in sinnvollen, der nötigen betriebswirtschaftlichen Aufrechterhaltung des Krankenhausbetriebs dienenden Grenzen hält, bleibt sie medizinethisch eher unproblematisch, mit Ausnahme der Gefahr einer Verselbständigung und Übersteigerung von Sparzwängen (‚Totsparen‘). *Kommerzialisierung* bedeutet etwas Spezifischeres als *Ökonomisierung*, nämlich das Ausrichten wirtschaftlicher Aktivitäten auf monetären Gewinn (bzw. Rendite) in einem Bereich, dem diese Ausrichtung bislang fremd war. Im Krankenhaus bedeutet Kommerzialisierung einen systematischen Anreiz, das Stellen von Diagnosen und Indikationen nicht nur am Wohl der Patienten, sondern auch an den für das Krankenhaus zu erwartenden Erlösen auszurichten“ (Arbeitsgruppe „Ökonomisierung“ 2020; Diskussionsprotokoll vom Marburger Symposion am 30. November 2019, S. 1).

7 Krobath (2010, S. 578) fasst den Klagenfurter Ansatz der OE vorläufig so zusammen: OE „ist die kontextsensitive Organisation ethischer Reflexion und Entscheidung als dauerhaft zu erneuernde Verständigungssysteme, Aushandlungsprozesse und kritische Differenzsetzungen in und zwischen Organisationen und Netzwerken über ‚das Gute‘ in unserer organisierten Gesellschaft, wie es innerhalb der betreffenden Organisation/Netzwerke, durch sie in weitere Umwelten und besonders für die von ihr Betroffenen wirksam werden sollte und könnte“.

8 Als „diskursive Macht“ bezeichne ich die Fähigkeit von Akteuren, mit argumentativen Mitteln die Kräfte zu verändern, die unseren Gründen dadurch zuwächst, dass Personen meinen, die Gründe, die sie haben, seien wirklich gute Gründe (vgl. Haugaard und Kettner 2020).

9 Mit Bezug auf das wichtige Problem einer aufschlussreichen Organisationstaxonomie, das leider nur spärlich adressiert wird in der breiten und vielfältigen organisationswissenschaftlichen Literatur (zu Schlüsselwerken dieser Literatur s. Kühl 2015; als Standard-Lehrbuch s. Schreyögg und Geiger 2015; zum *state of the art* s. laufend die Zeitschrift *Organization Studies (OS)*), sticht das Handbuch von Apelt und Tacke (2012) hervor.

10 Zur Begründung der Hypothese, dass die kommerzielle Überformung der Arzt-Patient-Interaktion das ärztliche Professionsethos in der Klinik in eine miserable Lage bringt, s. Kettner und Loer (2011).

11 Auch die entwicklungspsychologische Unterscheidung einer Reihe von Stufen des moralischen Urteilsvermögens konnte die, besonders im Forschungsprogramm der Diskursethik lange gehegte Hoffnung auf einen einzigen, allen anderen vernünftigen Positionen überlegenen moralischen Gesichtspunkt nicht einlösen (Kettner 2018).

12 Ich spreche im Folgenden generisch von „aktiven organisierten Sozialgebilden“, um konkrete Organisationen jeder Art, Institutionen, Gemeinschaften, Netzwerke im Blick zu behalten und die Engführung auf „soziale Systeme“, wie sie im Gefolge Niklas Luhmanns dogmatisiert wurde, zu vermeiden.

13 Beginnend mit Kets de Vries (2001, orig. 1980). Siehe auch Cygler und Sroka (2014).

14 Für einen Überblick innerhalb der Tradition der Frankfurter Schule vgl. Freyenhagen (2019). Rezente Überblicke bei Laitinen und Särkelä (2018), Honneth (2014, 1994).

15 Den wichtigen, aber vieldeutigen Begriff einer Funktion gebrauchen wir nicht im mathematischen Sinne, sondern in einem teleologischen (und z. B. für die Biologie unverzichtbaren) Sinne. Zur wissenschaftstheoretischen Klärung des teleologischen Funktionsbegriffs s. Allen und Neal (2020).

16 Da die Diagnostik von Funktionsdefizienzen und Miseren ohne begründete evaluative und normative Standards nicht möglich ist – das gilt mutatis mutandis schon für die medizinische und klinisch-psychologische Diagnostik (Hucklenbroich 2018) –, öffnet sich in der Theorie institutioneller Pathologie, dem Kern des Sozialpathologie-Paradigmas, ein Feld von Fragen, die nicht mit empirischen i. S. von quantitativen und qualitativen, alles Normative ausklammernden Methoden zu bearbeiten sind, sondern nur in der Ethik, verstanden als Teil einer kritischen Theorie der Gesellschaft (Kettner 2003).

17 Eine Systematisierung, die für meinen Vorschlag der Fokussierung klinischer OE auf Verantwortungsstörungen bedeutsam ist, hat der Betriebswirtschaftler Günter Ortmann ausgearbeitet. Ortmann (2011) präpariert an vielen Beispielen ein Repertoire von Mechanismen der Moralverdrängung in und durch Organisationen heraus, allerdings ohne die Implikationen für OE als angewandte Ethik auszuarbeiten.

## References

[CR3] Allen C, Neal J (2020) Teleological notions in biology. In: Zalta EN (Hrsg) The Stanford encyclopedia of philosophy (spring 2020 edition). https://plato.stanford.edu/archives/spr2020/entries/teleology-biology. Zugegriffen: 5. Jan. 2021

[CR1] Apelt M, Tacke V (2012). Handbuch Organisationstypen.

[CR2] Arbeitsgruppe „Ökonomisierung“ in der Akademie für Ethik in der Medizin (2020) Wie wär’s mit Widerstand? Ärztliche Handlungsoptionen gegen die Kommerzialisierung des Gesundheitswesens. Protokoll und Vorträge. http://www.ag-oekonomisierung.de/Download. Zugegriffen: 24. Nov. 2020

[CR4] Beauchamp T, Childress J (2019). Principles of biomedical ethics: marking its fortieth anniversary. Am J Bioeth.

[CR5] Cygler J, Sroka W (2014). Structural pathologies in inter-organizational networks and their consequences. Procedia Soc Behav Sci.

[CR7] Donaldson T (2009). Compass and dead reckoning: the dynamic implications of ISCT. J Bus Ethics.

[CR8] Frewer A, Fahr U, Rascher W (2008). Klinische Ethikkomitees. Chancen, Risiken und Nebenwirkungen.

[CR9] Freyenhagen F, Hammer E, Honneth A, Gordon PE (2019). Social pathology and critical theory. Routledge companion to the Frankfurt school.

[CR11] Gert B (1998). Morality. Its nature and justification.

[CR10] Gert B (2007). Common morality. Deciding what to do.

[CR12] Hall RT (2000). An introduction to healthcare organizational ethics.

[CR13] Haugaard M, Kettner M (2020). Theorising noumenal power.

[CR15] Heintel P, Krobath T, Heller A (2010). Organisation der Ethik. Ethik organisieren. Handbuch der Organisationsethik.

[CR14] Heubel F, Kettner M (2012). Lob der Profession. Ethik Med.

[CR17] Honneth A, Honneth (1994). Pathologien des Sozialen. Tradition und Aktualität der Sozialphilosophie. Pathologien des Sozialen. Die Aufgabe der Sozialphilosophie.

[CR16] Honneth A (2014). Die Krankheiten der Gesellschaft. Annäherung an einen nahezu unmöglichen Begriff. WestEnd 11.

[CR18] Hucklenbroich P (2018). Krankheit als theoretischer Begriff der Medizin: Unterschiede zwischen lebensweltlichem und wissenschaftlichem Krankheitsbegriff. J Gen Philos Sci.

[CR19] Jacobs K, Kettner M (2016). Zur Theorie sozialer Pathologien bei Freud, Fromm, Habermas und Honneth. IMAGO.

[CR6] Kets de Vries MFR (2001). Organizational paradoxes. Clinical approaches to management.

[CR26] Kettner M, Düwell M, Hübenthal C, Werner M (2002). Moral. Handbuch Ethik.

[CR25] Kettner M, Demirovic A (2003). Kritische Theorie und die Modernisierung des moralischen Engagements. Modelle kritischer Gesellschaftstheorie. Traditionen und Perspektiven der Kritischen Theorie.

[CR24] Kettner M, Frewer A, Fahr U, Rascher W (2008). Autorität und Organisationsformen Klinischer Ethikkomitees. Klinische Ethikkomitees. Chancen, Risiken und Nebenwirkungen.

[CR23] Kettner M, Kettner M, Koslowski P (2011). Organisationsethik im Krankenhaus – das nächste große Ding?. Wirtschaftsethik in der Medizin. Wie viel Ökonomie ist gut für die Gesundheit?.

[CR22] Kettner M, Maring M (2014). Wann haben wir ein moralisches Problem?. Bereichsethiken im interdisziplinären Dialog.

[CR21] Kettner M, Dörries A, Lipp V (2015). Spannungen zwischen Medizin und Ökonomie. Medizinische Indikation. Ärztliche, ethische und rechtliche Perspektiven. Grundlagen und Praxis.

[CR20] Kettner M, Rapic S (2018). Ohne Entwicklungslogik. Diskursethik und moralische Normativität diesseits von Kohlberg. Die Entwicklungslogik der Normativität. Probleme und Perspektiven.

[CR27] Kettner M, Loer T (2011). Das Wirkbündnis in der Arzt/Patient-Interaktion als moralischer Maßstab für Ökonomisierungsprozesse im Krankenhaus.

[CR28] Krainer L, Krobath T, Heller A (2010). Prozessethik als Widerspruchsmanagement. Zwischen theoretischen Prämissen und praktischen Hinweisen. Ethik organisieren. Handbuch der Organisationsethik.

[CR30] Krobath T, Krobath T, Heller A (2010). Zur Organisation ethischer Reflexion in Organisationen. Ethik organisieren. Handbuch der Organisationsethik.

[CR29] Krobath T, Heller A (2010). Ethik organisieren. Handbuch der Organisationsethik.

[CR31] Kühl S (2015). Schlüsselwerke der Organisationsforschung.

[CR32] Laitinen A, Särkelä A (2018). Analyzing conceptions of social pathology. Eight questions. Stud Soc Polit Thought.

[CR33] Neitzke G, Vollmann J, Schildmann J, Simon A (2009). Formen und Strukturen Klinischer Ethikberatung. Klinische Ethik. Aktuelle Entwicklungen in Theorie und Praxis.

[CR34] Neuhäuser C (2011). Unternehmen als moralische Akteure.

[CR35] Ortmann G, Haubl R, Möller H, Schiersmann C (2011). Moralverdrängung in und durch Organisationen. Positionen. Beiträge zur Beratung in der Arbeitswelt.

[CR36] Sabin JE (2016). How can clinical ethics committees take on organizational ethics? Some practical suggestions. J Clin Ethics.

[CR37] Scanlon T (2000). What we owe to each other.

[CR38] Schreyögg G, Geiger D (2015). Organisation. Grundlagen moderner Organisationsgestaltung. Mit Fallstudien.

[CR39] Siegler M (2019). Clinical medical ethics: its history and contributions to American medicine. J Clin Ethics.

[CR40] Stern (2019) Medizin für Menschen – Ärzte fordern Rückbesinnung auf Heilkunst statt Profit. https://www.stern.de/gesundheit/aerzte-appell-im-stern--die-titelgeschichte-zum-nachlesen-8902860.html. Zugegriffen: 24. Nov. 2020

[CR41] Suhonen R, Stolt M, Virtanen H, Leino-Kilpi H (2011). Organizational ethics. A literature review. Nurs Ethics.

[CR42] Woellert K (2019). Das Klinische Ethikkomitee: Ziele, Strukturen und Aufgaben Klinischer Ethik. Bundesgesundheitsblatt Gesundheitsforschung Gesundheitsschutz.

